# Serum miR-92a is Elevated in Children and Adults with Obstructive Sleep Apnea

**Published:** 2020-07-27

**Authors:** Brendan Gongol, Fenqing Shang, Ming He, Yingshuai Zhao, Weili Shi, Manli Cheng, John YJ. Shyy, Liuyi Wang, Atul Malhotra, Rakesh Bhattacharjee

**Affiliations:** 1 Division of Cardiology, Department of Medicine, University of California, San Diego, La Jolla, CA, USA; 2 Department of Cardiology, Xi’an First Hospital, 30 Fenxiang Road, Beilin District, Xi’an Shaanxi, P.R. China; 3 Department of General Medicine, Henan Provincial People’s Hospital, 7 Weiwu Road, Jinshui District, Zhengzhou Henan, P.R. China; 4 Division of Pulmonary, Critical Care, and Sleep Medicine, Department of Medicine, University of California San Diego, La Jolla, CA, USA; 5 Division of Respiratory Medicine, Department of Pediatrics, University of California San Diego, La Jolla, CA, USA

**Keywords:** Obstructive sleep apnea, Biomarkers, Micro RNA, Endothelial dysfunction

## Abstract

**Background:**

Obstructive Sleep Apnea (OSA) is a highly prevalent condition that is associated with several comorbidities including cardiovascular disease (CVD). Recent studies have revealed mixed results as to whether standard OSA therapy reverses CVD in adult patients. Thus, many advocate for earlier recognition of OSA induced CVD, as early as childhood, to prompt treatment antecedent to the onset of irreversible CVD. Here we investigated if the serum level of miR-92a, a known biomarker for CVD, can be used to identify patients with OSA in both children and adults.

**Methods:**

Consecutive snoring patients undergoing polysomnography were recruited for determination of circulating miR-92a, in addition to inflammatory and metabolic profiles. We assessed whether circulating miR-92a was associated with OSA severity.

**Results:**

Using two separate cohorts of adults (n=57) and children (n=13), we report a significant increase in the serum level of miR-92a in patients with severe OSA (p=0.021) and further demonstrate a significant correlation (Spearman rank correlation 0.308, p=0.010) with serum miR-92a levels and the apnea hypopnea index (AHI), a primary measure of OSA severity. Stepwise regression analysis revealed that serum miR-92a levels were independently associated with AHI (ß=0.332, p=0.003), age (ß=0.394, p=0.002) and LDL cholesterol levels (ß=0.368, p=0.004).

**Conclusion:**

Our study is the first to establish that miR-92a is a useful biomarker for OSA severity in both children and adults. Given the canonical role of miR-92a on endothelial dysfunction, miR-92a may be useful to identify early onset CVD in OSA patients or stratify patient CVD risk to identify those that may benefit from earlier OSA treatment.

## Introduction

Obstructive Sleep Apnea (OSA) is highly prevalent in both children and adults and defined by recurrent episodes of soft tissue impingement into the oral pharynx leading to an intermittent cessation of normal ventilation during sleep. As a consequence, patients with OSA experience episodic oxyhemoglobin desaturation, hypercapnia, frequent arousals during sleep, and subsequent sleep fragmentation. Epidemiological studies suggest that at least 10% of adults and 2–3% of the children have OSA with clinical consequences [1–3]. However, despite a lower reported prevalence rate of OSA in children compared to adults, it does remain one of the more common childhood diseases [4–7]. Moreover, given the near doubling of childhood obesity in the US [8], the observed prevalence of OSA has increased even further with reports of OSA prevalence in up to 6% of children [9–13].

While some have suggested that adult and pediatric OSA are different diseases, the impact of obesity on the phenotype of pediatric OSA has made these two conditions more similar than different [14]. Further, the findings from recent multicenter studies have failed to show an improvement in adult OSA outcomes following CPAP therapy [15] which have led many investigators to suggest that diagnosis and therapy should be initiated far earlier in the disease course i.e. before its irreversible consequences have occurred. Thus, we and others have proposed that the study of pediatric and adult OSA be harmonized to identify unifying biomarkers applicable to both patient populations.

Of note, OSA is associated with cardiovascular disease (CVD) in both children and adults. Longitudinal studies of adult patients with severe OSA have shown a three-fold increase in the risk of all-cause mortality, and a higher cardiovascular mortality at 18-year follow-up, respectively [16,17]. OSA is strongly associated with hypertension [18], myocardial ischemia [19], arrhythmias [20], ischemic stroke [21,22], such that the cumulative impact of OSA may account for the reported increases in both fatal and nonfatal cardiovascular events in adult patients [23,24].

In children, recent studies have shown that OSA is also associated with CVD [25]. Recent studies have confirmed that OSA independently leads to blood pressure dysregulation, hypertension [26–28], and left ventricular dysfunction in young children [29]. Of relevance to this study, OSA is also associated with endothelial dysfunction (ED) [30–32] with OSA treatment reversing ED in non-obese children [30].

The vascular endothelium, which lines the luminal arterial wall, is an important tissue type that governs vascular health or diseases such as atherosclerosis [33,34]. As the frontline of vascular integrity, ED represents a very early manifestation of CVD [33,34] and thus may be observed in both adult and pediatric populations. Parenthetically, ED associated with OSA is likely the result of initiation and propagation of inflammatory responses within the vasculature [35] such that assessment of ED should identify patients at high risk of OSA associated CVD.

The study of extracellular microRNA (miRs) have gained considerable traction for the identification of ED. Extracellular miRs are 21–23 nucleotide non-coding nucleic acids found in serum, plasma or other body fluids [36–38]. In studies of CVD, changes in extracellular levels and activities of several miRs have been linked to acute myocardial infarction and heart failure [39].

Our laboratory has shown an increased expression of miR-92a within serum samples isolated from patients with coronary artery disease or chronic kidney disease [40,41]. We have also shown that oxidative stress from angiotensin II, athero-prone flow, and oxidized low-density lipoprotein (oxLDL) stimulation induce miR-92a in endothelium signifying that miR-92a is a biologically plausible biomarker of ED. In animal studies, miR-92a is associated with dyslipidemia [42] and sterol regulatory element-binding protein 2 (SREBP2), a key transcription factor involved in activation of genes in cholesterol biosynthesis, appears to also induce miR-92a [40,43] implying that an additional mechanism for miR-92a mediated CVD is through dyslipidemia. However, the link between OSA and dyslipidemia remains controversial, emphasizing the need for further study.

Given the association of OSA with both CVD and dyslipidemia [44–47], we therefore hypothesize that miR-92a is an important biomarker of OSA severity, independent of several confounding variables including age. Since miR-92a is known to illicit ED thereby indicating early onset CVD, we speculate that increased miR-92a expression may be a valid biomarker of OSA associated CVD in both clinical adult and pediatric OSA patients.

## Materials and Methods

### Patient selection and examination

#### Adults:

Research was approved by the institutional ethics committee of Xi’an Number One hospital (IRB# xadyyy2018028). Adult patients were recruited and written consent was obtained prior to enrollment. Patients diagnosed with OSA met the published guidelines for the diagnosis and treatment of obstructive sleep apnea syndrome [48]. Patients were excluded if associated with pulmonary disease, e.g., chronic obstructive pulmonary disease, bronchial asthma, interstitial pneumonia, chronic respiratory failure. Patients who have received continuous positive airway pressure ventilation or related surgical treatment were also excluded. Other exclusions included patients with severe cardiovascular and cerebrovascular diseases, e.g., coronary heart disease, stroke, type 2 diabetes, etc.; moderate to severe liver or kidney dysfunction; chronic insomnia; acute or chronic infectious diseases; tumors, hematopathy, trauma, autoimmune diseases (e.g., rheumatoid arthritis, systemic lupus erythematosus, inflammatory bowel disease); and finally, patients with mental health disorders.

#### Children:

Study procedures were approved by the Institutional Review Board at University of California, San Diego (IRB# 170408). From February 2017-August 2019, consecutive children were approached for recruitment for participation in this study. Children with craniofacial syndromes, Down syndrome or other defined genetic abnormalities, neuromuscular or other congenital disorders or other systemic diseases were excluded. Children with identified central sleep apnea (central apnea index>5 event/hr) were also excluded from this study. Finally, children with hypertension, uncontrolled diabetes, or history of smoking including vaping (any smoking within the prior 6 mos. or >5 pack years total) were excluded.

#### miR isolation and detection

miR isolation was performed using Trizol LS reagent according to the manufacturer’s instructions. 500 uL of patient serum was used for the isolation and analysis of miR levels and 2 nM reference *Caenorhabditis elegans* miR-39 (cel-miR-39) was added to the serum prior to the addition of three volumes of Trizol LS reagent. Following isolation, miRs were reverse transcribed with TaqMan™ MicroRNA Reverse Transcription Kit (Applied Biosystems. Cat# 4366597) according to the manufacturer’s instructions. Following traverse transcription, the levels of miR-92a were determined via qPCR using the Applied Biosystems™, TaqMan™ Universal Master Mix II, no UNG (Cat# 4440040). Expression levels were determined using the Δ−Δct method using cel-miR-39 spike in as a reference. Fold change values were then determined by dividing the expression level with the average of the expression level of the primary snoring for each site.

#### Polysomnography

Sleep studies were performed in adults were performed at Xi’an Number One hospital using the somnolab 2 system and in children at Rady Children’s Hospital using Nihon Koden (Tokyo, Japan) PSG equipment and software. The following parameters were measured: chest and abdominal wall movement by inductance plethysmography, heart rate by electrocardiography, air flow using nasal pressure and oronasal thermistor. In children, additional monitoring using a side-stream end-tidal capnograph (Nihon Koden) to provide breath-by-breath assessment of end-tidal carbon dioxide levels. Arterial pulse oxygen saturation (SpO_2_) was assessed by pulse oximetry, with simultaneous recording of the pulse waveform. The bilateral electrooculogram, 8 channels of electroencephalogram (2 frontal, 2 occipital, 2 temporal and 2 central leads), chin and anterior tibial electromyograms, and analog output from a body position sensor were also monitored. PSG was scored by board registered technologists (RPSGT) and interpreted by board certified sleep physicians according to the 2017 American Academy of Sleep Medicine Manual for the Scoring of Sleep and Related Events [49].

We reviewed all PSGs for the following parameters: apnea hypopnea index (AHI) (total number of apneas and hypopneas per hour of total sleep time (TST); the oxygen desaturation index (ODI) was defined as the number of desaturation events ≥ 3% per hour of TST; the lowest SaO_2_ or oxygen saturation nadir was the lowest observed oxygen saturation during sleep; and the %TST O_2_<90% was the percentage of TST with an observed oxygen saturation below 90%. In children, OSA was defined if the obstructive AHI was greater than 1.5 events/hr; mild OSA consisted of obstructive AHI from 1.5–5 events/hr, moderate OSA with an obstructive AHI from 5–10 events/hr, and severe OSA with an obstructive AHI>10 events/hr. In adults, OSA was defined if the obstructive AHI was greater than 5 events/hr; mild OSA consisted of obstructive AHI from 5–15 events/hr, moderate OSA with an obstructive AHI from 15–30 events/hr, and severe OSA with an obstructive AHI>30 events/hr.

#### Statistical analysis

Results are presented as means ± SD, unless stated otherwise. All numerical data were subjected to statistical analysis using paired t tests or analysis of variance followed by post hoc tests (Tukey) as appropriate. Chisquare analysis was performed on categorical data concerning demographic characteristics of the various groups. Finally, univariate correlation analyses (Spearman rank) and multivariate linear regression modelling were performed to explore the relationships between sets of variables. Statistical analyses were performed using SPSS version 26.0 (SPPS Inc., Chicago, IL). For all comparisons, a two-tailed P value less than 0.05 was considered to define statistical significance.

## Results

### Subject characteristics

In total, 84 patients were recruited (58 adults, 26 children). Successful identification of miR-92a from serum occurred in 70 patients (57 adults, 13 children). In fourteen patients (1 adult, 13 children) levels of miR-92a was not quantifiable due to extraction error. A comparison of demographic, polysomnographic, inflammatory and metabolic markers of included versus excluded children did not reveal any statistically significant differences apart from total cholesterol levels (p=0.048) (Supplementary table). The average age ± SD of patients was 40.6 ± 17.9 years (range: 7.3–81.0 years). There were 17 females (24%). Of the 70 patients, 34 were identified as obese based on BMI>30 kg/m^2^ for adults, or based on BMI>95^th^ percentile for children (Table 1).

From polysomnography data, the average ± SD apnea hypopnea index was 20.4 ± 22.1 events/hr (Table 1). In adults, where AHI>30 events/hr was used to define severe OSA, 17 of 57 adults (30%) were found to have severe OSA. In children, using criteria where AHI>10 events/hr to define severe OSA, 4 of 13 children (31%) had severe OSA (Table 1).

### Serum investigations

**Figure 1.**
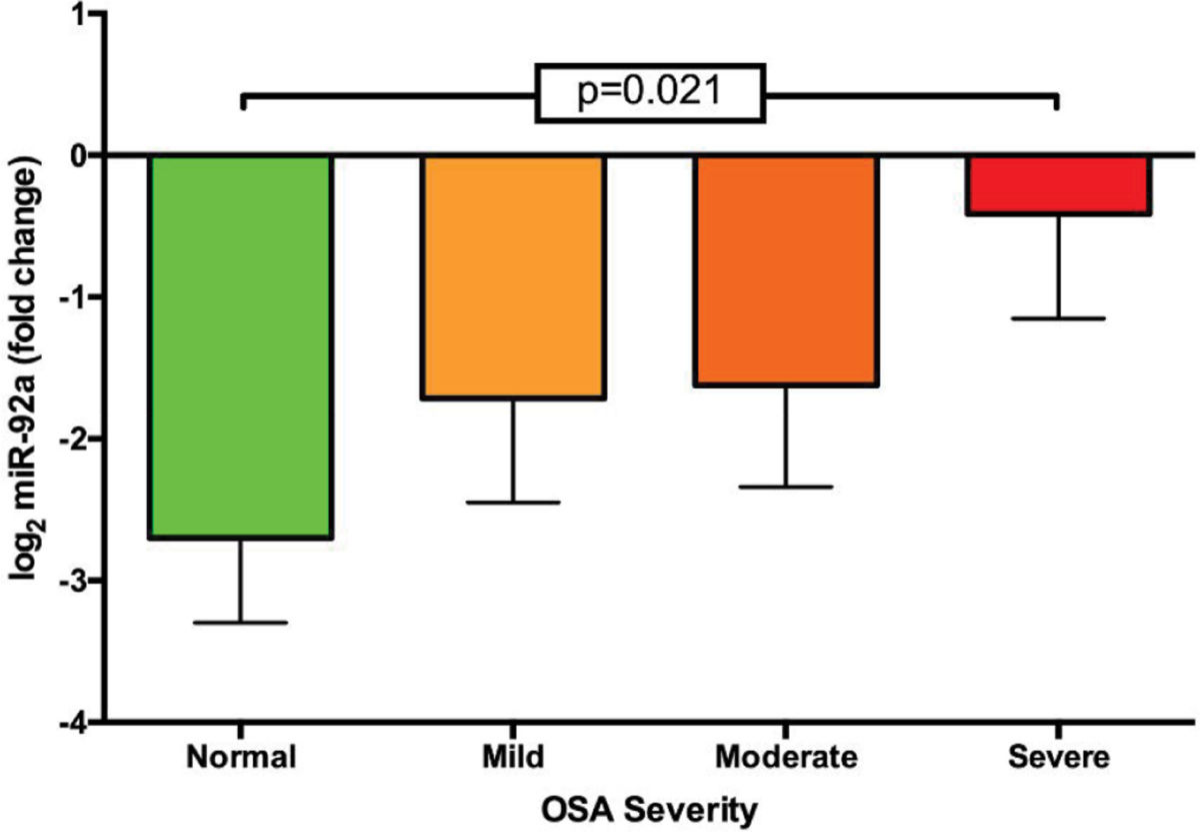
Change in miR-92a expression across different OSA severities comparison of change in miR-92a expression across OSA severity groups. Severe OSA patients had significant elevations in miR-92a expression compared to patients with normal sleep studies (p=0.021). Data expressed as mean ± SEM.

Summary of serum data for metabolic and hepatic biomarkers can be found in Table 2. Comparing change in miR-92a levels using the delta CT method revealed a stepwise increase in logarithmic miR-92a across all OSA severities; with severe OSA having the largest increase in log_2_ miR-92a expression (p=0.021 from primary snoring patients) (Figure 1).

**Figure 2.**
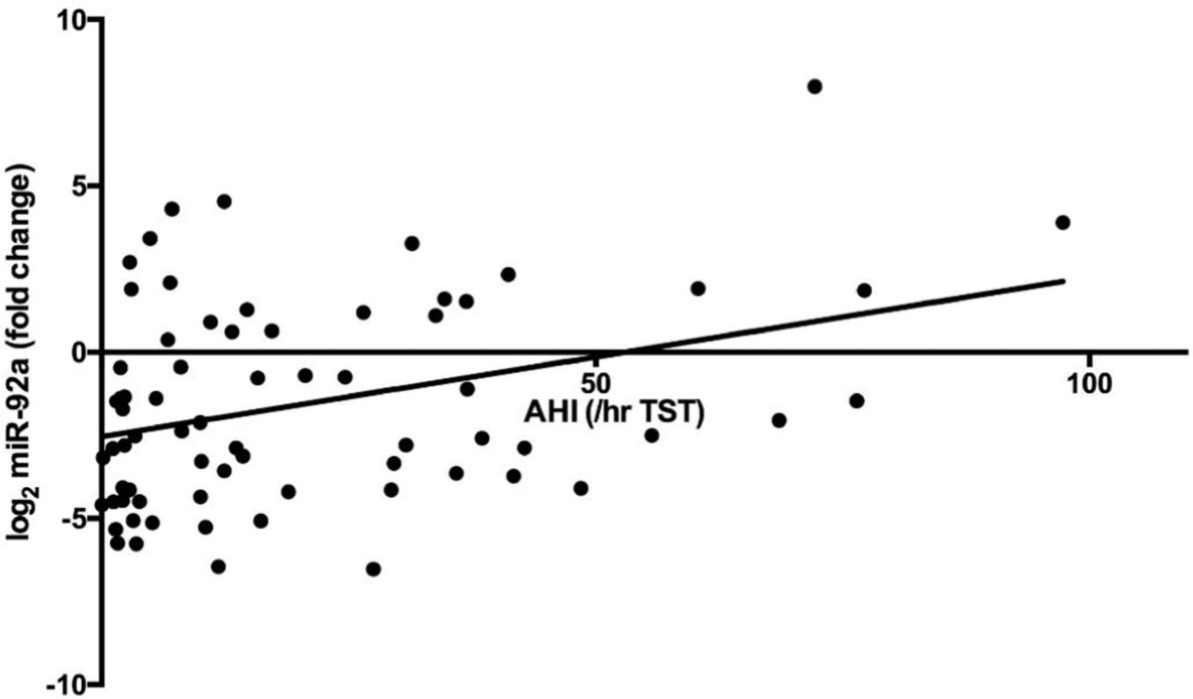
Relationship between the apnea hypopnea index and change in miR-92a expression. Pearson correlation 0.346, p=0.003. (TST-total sleep time).

Evaluating univariate correlation of all serum biomarkers and polysomnography parameters with delta log_2_ miR-92a revealed that AHI (Spearman rank correlation 0.308, p=0.010), and ODI (Spearman rank correlation 0.277, p=0.037) had statistically significant associations (Figure 2). Of inflammatory or metabolic markers associated with delta log_2_ miR-92a, only LDL cholesterol (Spearman rank correlation 0.270, p=0.025) and C-reactive protein (CRP) (Spearman rank correlation 0.262, p=0.030) concentrations were significantly associated with delta log_2_ miR-92a.

Despite findings from the univariate analysis, we assessed the association of AHI and delta log_2_ miR-92a using a stepwise reverse linear regression model, in which, in addition to AHI, LDL cholesterol, CRP (based on borderline associations from univariate analysis), and other relevant biological covariates such as age, BMI, and sex (Table 3) were controlled for. Again, AHI remained significantly associated with delta log_2_ miR-92a (β=0.332, p=0.003). In this model, age (β=0.394, p=0.002) and LDL Cholesterol (β=0.368, p=0.004) also emerged as significantly associated with delta log_2_ miR-92a, while sex, BMI and CRP were unassociated.

## Discussion

To our knowledge, our study is the first addressing OSA consequences using both pediatric and adult cohorts. Our study found that independent of age, BMI, and sex, the presence of OSA was associated with an increase in miR-92a expression, and in a severity dependent fashion. We believe that miR-92a may represent an important biomarker for OSA as it provides both mechanistic insights and a potential therapeutic target for subsequent studies.

Our selection to investigate miRNAs and specifically miR-92a expression was deliberate. Elevation of miR-92a has been found to prevent angiogenesis [50] impairing normal vascular responses to conditions such as ischemia. Further, miR-92a appears to be highly involved in the activation of the vascular endothelium in atherosclerotic plaque formation. Inhibition of miR-92a expression was also shown to reduce endothelial inflammation, and altered atherogenesis in a rodent model of atherosclerosis [51]. Oxidized LDL, which predisposes to atherosclerosis, enhances upregulation of miR-92a in endothelial cells through activation of the signal transducer and activator of transcription 3 (STAT3), promoting inflammation in endothelial cells and increased adhesiveness to circulating monocytes, a canonical component of atherosclerosis. This noncoding miRNA appears to also regulate endothelial cell activation by modulation of several transcription factors including Kruppel-like factor 2 (KLF2), Kruppel-like factor 4 (KLF4), Nuclear Factor – kappa B (NF-кB) and the suppressor of cytokine signaling 5 (SOCS5) [51–53]. Taken together, the effects of miR-92a in endothelial activation are likely centrally involved in ED and atherosclerosis development, rendering miR-92a as a useful biomarker for CVD. In this context, not surprisingly, miR-92a has been found to be elevated in patients with acute coronary syndrome and with unstable angina [54–56].

ED represents a very early manifestation of CVD [33] such that assays for ED, including measuring miR-92a levels, may measure pre-clinical CVD, a stage when CVD is most amenable to intervention. As such, a biomarker that identifies pre-clinical CVD is of paramount importance as it may be clinically actionable. The ideal biomarker of vascular susceptibility should also have sensitivity and specificity for disease, be a robust metric of disease severity, and should improve following therapy for disease [57–59]. Further, it would be on a causal pathway to important disease outcomes giving its scientific credibility.

Here we present that miR-92a may be an ideal biomarker for OSA and possibly OSA induced CVD. OSA has been shown to induce ED in both children [30–32] and in adults [60]. Moreover, the treatment of OSA in young children and in adults leads to a reversal of ED [30,61]. Given the upregulation of miR-92a with severe OSA and its significant association with the AHI implies that OSA promotes ED through signaling cascades downstream of miR-92a. Thus, miR-92a also provides a possible mechanistic link between OSA and atherosclerosis. Our multivariate model revealed a significant association between miR-92a and LDL cholesterol which is expected given that *in vivo* studies have demonstrated that LDL cholesterol promotes miR-92a upregulation [62]. Notwithstanding, the association of OSA and miR-92a was independent of LDL cholesterol and BMI/obesity status, factors both known to increase the risk of atherosclerosis independently, strengthening the utility of miR-92a as a biomarker for OSA induced CVD.

However, it is important to acknowledge the limitations of our study. First, the study is limited by a small sample size, particularly in females and in children. Nonetheless, the inclusion of children further strengthened the statistical relationship, and also confirmed that utility of this biomarker across a broad age group. Future studies will aim to assess miR-92a in larger cohorts of both adults and children with an aim to increase the number of females in our cohort. A larger sample size could better delineate other factors that are associated with miR-92a beyond the AHI. Second, due to the logistics of our study, we were unable to assess for endothelial function specifically in patients using techniques such as flow mediated dilation; these studies could further evaluate the relationship between OSA, ED and miR-92a. However, using two diverse cohorts across separate geographical locations certainly adds to the external validity of this biomarker. Finally, we did not assess the effect of OSA treatment on miR-92a expression. While studies assessing treatment of OSA using continuous positive airway pressure (CPAP) are complex, this approach would better delineate the bidirectional relationship between OSA and miR-92a levels. However, we strongly believe that pilot data such as those which we report here are necessary before the risk and expense of a clinical trial can be justified or even properly designed.

## Conclusion

The identification of a miR-92a as a valid biomarker in identifying OSA may also help to classify patients at risk for CVD. Knowing that increased miR-92a implies poor vascular health, this finding could help identify OSA patients that are susceptible to CVD. Recent focus has occurred regarding phenotypic clustering in OSA patients with only subsets being at major cardiovascular risk. Such studies have not included pediatric patients systematically to date. We would be supportive of use of biomarkers (such as miR-92a) to classify patients for research studies e.g. to enroll high risk patients into clinical trials with appropriately focused endpoints. Clinically, assaying for miR-92a in OSA patients could help clinicians identify at risk patients that could lead to strategies such as earlier aggressive treatment or determining which patients require closer monitoring and follow up.

## Supplementary Material

Sup file

## Figures and Tables

**Table 1.** Demographic summary of study population (N=70).

Age (y)	Number (%)	Mean ± SD		Range
		
		40.6 ± 17.9		(7.3–81)

		**Sex**		

Female	17 (24%)	--		--

Male	53 (76%)	--		--

BMI (kg/m^2^)	--	29.2 ± 5.3		(16.6–46.4)

		**Obesity Status**		

Obese	34 (49%)	--		--

NonObese	36 (51%)	--	-	-

		**Polysomnography Findings**		

AHI (/hr TST) {n=70)	--	20.4 ± 22.1		(0.0–97.3)

ODI (/hr TST) {n=57)	--	19.6 ± 21.0		(0.0–103.4)

%TST O2 Sat<90% (%) {n=38}	--	7.7 ± 17.9		(0.0–91.5)

O2 Sat Nadir (%) {n=38}	--	78.9 ± 11.9		(46–95)

		**OSA Severity**		

**Parameters**	**Normal**	**Mild**	**Moderate**	**Severe**

UCSD	2 (15.4%)	4 (28.6%)	3 (23.1%)	4 (28.6%)

XIAN	16 (28.1%)	16 (28.1%)	11 (19.3%)	21 (36.8%)

**Table 2.** Serum/plasma markers of cardiometabolic disease (N=70).

Variables	Mean ± SD	Range
Insulin (mU/mL)	18.1 ± 19.0	(2.1–110.0)
CRP*(mg/L)	2.0 ± 1.7	(0.0–9.0)
Glucose (mg/dL)	91.8 ± 16.0	(70.4–156.4)
AST (U/L)	27.0 ± 15.8	(6.0–110.0)
ALT (U/L)	33.5 ± 27.9	(8.0–201.0)
Total Cholesterol (mg/dL)	89.4 ± 34.4	(40.5–210.0)
Triglyceride (mg/dL)	52.7 ± 56.1	(8.8–421.0)
HDL (mg/dL)	22.6 ± 8.2	(12.6–48.4)
LDL*(mg/dL)	54.0 ± 24.3	(16.0–152.2)
Cholesterol:HDL ratio	1.6 ± 13	(1.6–7.1)
Log2 miR-92a (fold change)	1.6 ± 3.1	(−6.5–8.0)

* n=69

**Table 3.** Multivariate linear regression analysis with Log_2_ MIR-92A.

Variables	Standardize Beta	p Value
AHI (/hr TST)	0.324	0.022
Age (y)	0.363	0.006
Sex	0.079	0.49
BMI (kg/m^2^)	−0.147	0.205
LDL * (mg/dL)	0.338	0.011
CRP*(mg/L)	0.092	0.517

* n=69

## Abbreviations:

OSA: Obstructive Sleep Apnea
CVD: Cardiovascular Disease
ED: Endothelial Dysfunction
miR: Micro RNA
AHI: Apnea Hypopnea Index
ODI: Oxygen Desaturation Index
TST: Total Sleep Time
O_2_ Sat: Oxygen Saturation
%TST O_2_<90%: Percent total sleep time with Oxygen Saturation Less than 90%
BMI: Body Mass Index
LDL: Low Density Lipoprotein
HDL: High Density Lipoprotein
CRP: C-Reactive Protein
AST: Aspartate Transaminase
ALT: Alanine Transaminase
