# Supplementary material for: Serum miR-92a is Elevated in Children and Adults with Obstructive Sleep Apnea
Source: J Mol Biomark Diagn. Author manuscript; Available in PMC 2021 Jan 7. (PMC7789821)
Supplement: Sup file [file NIHMS1652503-supplement-Sup_file.docx]

| **Comparison of Included and Excluded Children** | | | |
| --- | --- | --- | --- |
| **Variable** | **Included Children (n=13)** | **Excluded Children (n=13)** | **p Value** |
|  | **Mean ± SD** | **Mean ± SD** |  |
| Age (y) | 12.42 ± 3.54 | 11.42 ± 3.51 | 0.476 |
| **Sex** | | | |
| Female | 5 | 7 | 0.348 |
| Male | 8 | 6 |  |
| BMI (kg/m^2^) | 31.05 ± 7.21 | 27.32 ± 7.35 | 0.204 |
| **Obesity Status** | | | |
| Obese | 12 | 10 | 0.297 |
| Non-obese | 1 | 3 | - |
| AHI (/hr) | 17.01 ± 20.97 | 15.50 ± 28.17 | 0.878 |
| ODI (/hr) | 12.61 ± 15.30 | 8.09 ± 17.52 | 0.49 |
| % TST O2 Sat <90 (%) | 1.59 ± 2.57 | 1.91 ± 6.67 | 0.875 |
| O2 Sat nadir (%) | 85.23 ± 8.93 | 89.00 ± 12.00 | 0.373 |
| Insulin (mU/mL) | 31.15 ± 26.43 | 15.06 ± 10.04 | 0.051 |
| CRP*(mg/L) | 3.02 ± 2.77 | 2.22 ± 2.08 | 0.418 |
| Glucose (mg/dL) | 97.85 ± 13.57 | 94.85 ± 8.74 | 0.509 |
| AST (U/L) | 40.38 ± 21.61 | 29.00 ± 5.26 | 0.077 |
| ALT (U/L) | 60.08 ± 45.96 | 37.31 ± 14.62 | 0.102 |
| Total Cholesterol (mg/dL) | 141.46 ± 33.08 | 163.92 ± 20.53 | 0.048 |
| Triglyceride (mg/dL) | 120.31 ± 95.54 | 122.23 ± 47.11 | 0.949 |
| HDL (mg/dL) | 34.85 ± 7.49 | 39.46 ± 7.38 | 0.127 |
| LDL**(mg/dL) | 86.62 ± 29.44 | 100.31 ± 19.02 | 0.177 |
| Cholesterol:HDL ratio | 4.16 ± 1.03 | 4.34 ± 1.18 | 0.672 |
